# A Novel Poly-Naphthol Compound ST104P Suppresses Angiogenesis by Attenuating Matrix Metalloproteinase-2 Expression in Endothelial Cells

**DOI:** 10.3390/ijms150916611

**Published:** 2014-09-19

**Authors:** Yi-Ling Ma, Shih-Wei Lin, Hua-Chang Fang, Kang-Ju Chou, Youn-Shen Bee, Tian-Huei Chu, Ming-Chi Chang, Wen-Tsan Weng, Chang-Yi Wu, Chung-Lung Cho, Ming-Hong Tai

**Affiliations:** 1 Department of Biological Sciences, National Sun Yat-Sen University, Kaohsiung 804, Taiwan; E-Mails: ylma@vghks.gov.tw (Y.-L.M.); cywu@mail.nsysu.edu.tw (C.-Y.W.); 2 Division of Nephrology, Kaohsiung Veterans General Hospital, Kaohsiung 813, Taiwan; E-Mails: hcfang@vghks.gov.tw (H.-C.F.); kjchou@vghks.gov.tw (K.-J.C.); 3 National Sun Yat-sen University and Academia Sinica Doctoral Degree Program in Marine Biotechnology, National Sun Yat-Sen University, Kaohsiung 804, Taiwan; E-Mail: shiwey0214@gmail.com; 4 Department of Ophthalmology, Kaohsiung Veterans General Hospital, Kaohsiung 804, Taiwan; E-Mail: ysbee@vghks.gov.tw; 5 Institute of Biomedical Sciences, National Sun Yat-Sen University, Kaohsiung 804, Taiwan; E-Mail: skbboyz0817@gmail.com; 6 Division of Colorectal Surgery, Kaohsiung Veterans General Hospital, Kaohsiung 813, Taiwan; E-Mail: mcchang@vghks.gov.tw; 7 Institute of Basic Medical Sciences, National Cheng Kung University, Tainan 701, Taiwan; E-Mail: bpvincent@gmail.com; 8 Center for Stem Cell Research, Kaohsiung Medical University, Kaohsiung 807, Taiwan

**Keywords:** ST104P, angiogenesis, Lewis lung carcinoma, matrix metalloproteinase-2 (MMP-2)

## Abstract

Angiogenesis, the process of neovascularization, plays an important role in physiological and pathological conditions. ST104P is a soluble polysulfated-cyclo-tetrachromotropylene compound with anti-viral and anti-thrombotic activities. However, the functions of ST104P in angiogenesis have never been explored. In this study, we investigated the effects of ST104P in angiogenesis *in vitro* and *in vivo*. Application of ST104P potently suppressed the microvessels sprouting in aortic rings *ex vivo*. Furthermore, ST104P treatment significantly disrupted the vessels’ development in transgenic zebrafish *in vivo*. Above all, repeated administration of ST104P resulted in delayed tumor growth and prolonged the life span of mice bearing Lewis lung carcinoma. Mechanistic studies revealed that ST104P potently inhibited the migration, tube formation and wound closure of human umbilical endothelial cells (HUVECs). Moreover, ST104P treatment inhibited the secretion and expression of matrix metalloproteinase-2 (MMP-2) in a dose-dependent manner. Together, these results suggest that ST104P is a potent angiogenesis inhibitor and may hold potential for treatment of diseases due to excessive angiogenesis including cancer.

## 1. Introduction

Angiogenesis is the process by which new capillary blood vessels sprout from pre-existing vessels and occurs during many physiological and pathological conditions, such as tissue repair, fetal development, female reproductive cycle, tumor progression and metastasis. It is an essential step in both tumor growth and metastases. Since Folkman *et al.* first proposed the hypothesis that tumor growth is dependent on angiogenesis, inhibition of tumor angiogenesis has been extensively explored as a novel anti-tumor strategy in recent decades [1,2,3,4,5]. Angiogenesis is involved in multiple interactions between endothelial cells, other surrounding cells, extracellular matrix (ECM), and angiogenic growth factors. It is relevant to the proliferation, migration and tube formation of endothelial cells and degeneration and remodeling of extracellular matrix [6,7]. Steps during angiogenesis include degradation of the basement membrane surrounding an existing vessel, migration and proliferation of endothelial cells into the new space, maturation, differentiation, and adhesion of the endothelial cells to each other, and lumen formation. The process is tightly regulated by the balance between angiogenic stimulators and inhibitors. So far, a number of angiogenic inhibitors derived from different endogenous proteins or chemicals have been reported to inhibit angiogenesis *in vitro* and *in vivo* [8,9,10,11,12,13].

Matrix metalloproteinases (MMPs) are a broad family of zinc-binding endopeptidases that play a key role in ECM degradation associated with tumor cell invasion, metastasis and angiogenesis. In particular, MMP-2 and MMP-9 play an important role in the angiogenic responses in endothelial cells [14,15]. A vast number of synthetic MMP inhibitors (MMPIs) have been developed in recent years to target MMPs, trying to control their enzymatic activities in abnormal bio-processes [16]. Therefore, MMP-2 and MMP-9 have been the most investigated factors for their role in angiogenesis.

**Figure 1:**
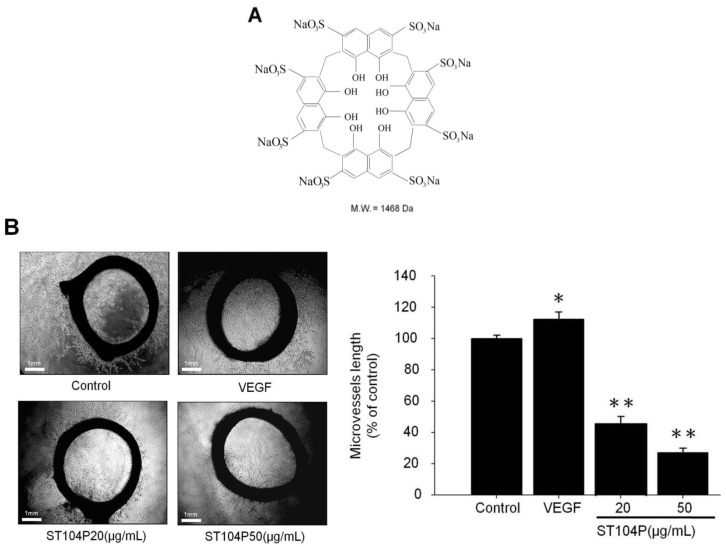
Effect of ST104P on angiogenesis *ex vivo*. (**A**) The chemical structure of ST104P; and (**B**) Rat aortic segments were placed into the Matrigel-covered wells andtreated with phosphate-buffered saline (PBS) or ST104P of the indicated dose for six days. Representative photographs of vessel sprouting were recorded and analyzed statistically (20× magnification. Scale bars = 1 mm). Data were mean ± standard error of the mean (SEM) of triplicate experiments. Asterisks indicate statistical significance *versus* control. * *p* ˂ 0.05 and ** *p* ˂ 0.01.

ST104P (a tetrameric cyclic compound of 4,5-dihydroxynaphthalene-2,7-disulfonic acid linked by methylene bridges) is a synthetic polysulfated-cyclo-tetrachromotropylene macrocyclic compound containing four naphthalene units in its cyclic structure (Figure 1A). Previous studies indicated that ST104P exhibits anti-viral and anti-thrombotic function with marginal cellular toxicity [17,18,19]. However, ST104P has never been indicated as an anti-angiogenic agent for treatment of diseases caused by or in association with undesirable angiogenesis, including cancer. It is therefore a subject of interest of the present investigation to provide a composition comprising ST104P exhibiting remarkable anti-angiogenic activity suitable for cancer therapy. To investigate the effect of ST104P on angiogenesis, we examined how this compound regulates endothelial functions and the underlying mechanism. In this study, we evaluated the effects of ST104P in animal models and cultured endothelial cells, and provided evidence regarding the influence of ST104P on endothelial cell functions *in vitro* and *in vivo*. Our results clarified the anti-angiogenic mechanisms of ST104P, attenuated proliferation, migration, tube formation and inhibited the secretion and expression of matrix metalloproteinase-2 (MMP-2) of cultured endothelial cells.

## 2. Results and Discussion

### 2.1. ST104P Suppressed the Microvessels Sprouting in Organotypic Aortic Rings

To evaluate the effect of ST104P on angiogenesis, we employed the organotypic aortic ring assay, which offers qualitative as well as quantitative measurement for both pro-angiogenic and anti-angiogenic factors. It was found that application of ST104P (at 20, 50 and 100 μg/mL) significantly inhibited microvessel sprouting (by approximately 50%, 70% and 97% of control, respectively; Figure 1B). Thus, these findings implicate the potential of ST104P in suppressing neovascularization *ex vivo*.

### 2.2. ST104P Perturbed the Vessels’ Development in Transgenic Zebrafish

**Figure 2:**
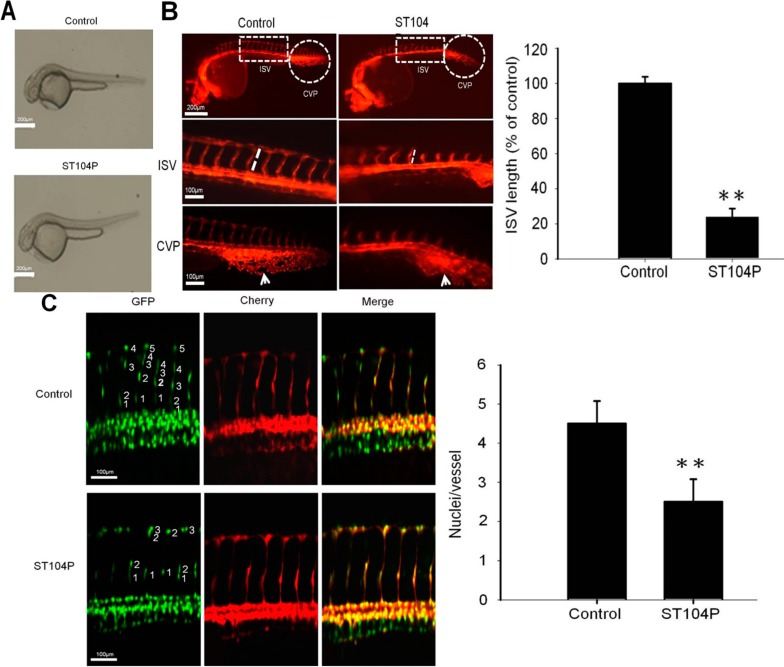
Effect of ST104P on vessel development in transgenic zebrafish models. The influence of ST104P (100 μg/mL) application on blood vessel development in *Tg(kdrl:mCherry)^ci5^* × *Tg(fli1a:negfp)^y7^* zebrafish embryos were analyzed at various time intervals. (**A**) Bright-field images of ST104P-treated embryos (Scale bars = 200 µm); (**B**) Fluorescence microscopy analysis of ST104P-treated embryos at 30 h post fertilization (hpf). **Upper** panels show the anatomical locations of the intersegmental vessels (ISV) and caudal vein plexus (CVP) for observation (50× magnification; scale bars = 200 µm); **Middle** panel shows the measurements of ISV length (corresponding to the boxed area in the **upper** panel) in control and ST104P embryos. The sprouting length of ISV in embryos (*n* = 10 per group) was analyzed at 30 hpf (100× magnification; scale bars = 100 µm). Data were mean ± SEM of triplicate experiments; **Bottom** panels highlight the morphology and neovascularization in CVP morphology as indicated by arrows; and (**C**) Quantification of endothelial number and migration in ISVs of ST104P-treated in transgenic *Tg(kdrl:mCherry)^ci5^* × *Tg(fli1a:negfp)^y7^* zebrafish. In this transgenic line, endothelial cells were labeled with green nuclei by *negfp* expression while the vessels were labeled with red by cytoplasmic mCherry expression. Images of ST104P-treated embryos were recorded at 48 hpf (200× magnification; scale bars = 100 µm). The number of endothelial cells on each vessel was quantified by counting the green nuclei on each red dorsal longitudinal anastomotic vessel (DLAV). Data were mean ± SEM of triplicate experiments. Asterisks indicate statistical significance *versus* control. ** *p* ˂ 0.01.

To further investigate the anti-angiogenic efficacy of ST104P, we employed *Tg(kdrl:mCherry)^ci5^* × *Tg(fli1a:negfp)^y7^* zebrafish embryos for easy monitoring of neovascularization in the intersegmental vessels (ISV) and caudal vein plexus (CVP) [20]. Application of ST104P elicited no obvious defect in gross morphology of zebrafish embryos (Figure 2A). However, ST104P treatment prominently perturbed ISV formation such that the sprouting length of ISV in ST104P-treated zebrafish was significantly shorter than control by about 70% (Figure 2B). Similarly, the CVP in ST104P-treated embryos were diffused and transformed while control siblings displayed a clear CVP network with spaces between capillaries. By counting the *negfp*-positive cells, imaging analysis revealed that ST104P treatment significantly decreased the number of endothelial cells on the dorsal longitudinal anastomotic vessel (DLAV; 2.5 ± 0.58) compared with that of control groups (4.5 ± 0.5; Figure 2C). This could be the consequence of attenuated migration and proliferation of endothelial cells by ST104P. Together, these results indicate that ST104P exerts anti-angiogenic activities *in vivo*.

### 2.3. Injection of ST104P Suppressed Tumor Growth and Prolonged Survival in Mice

**Figure 3:**
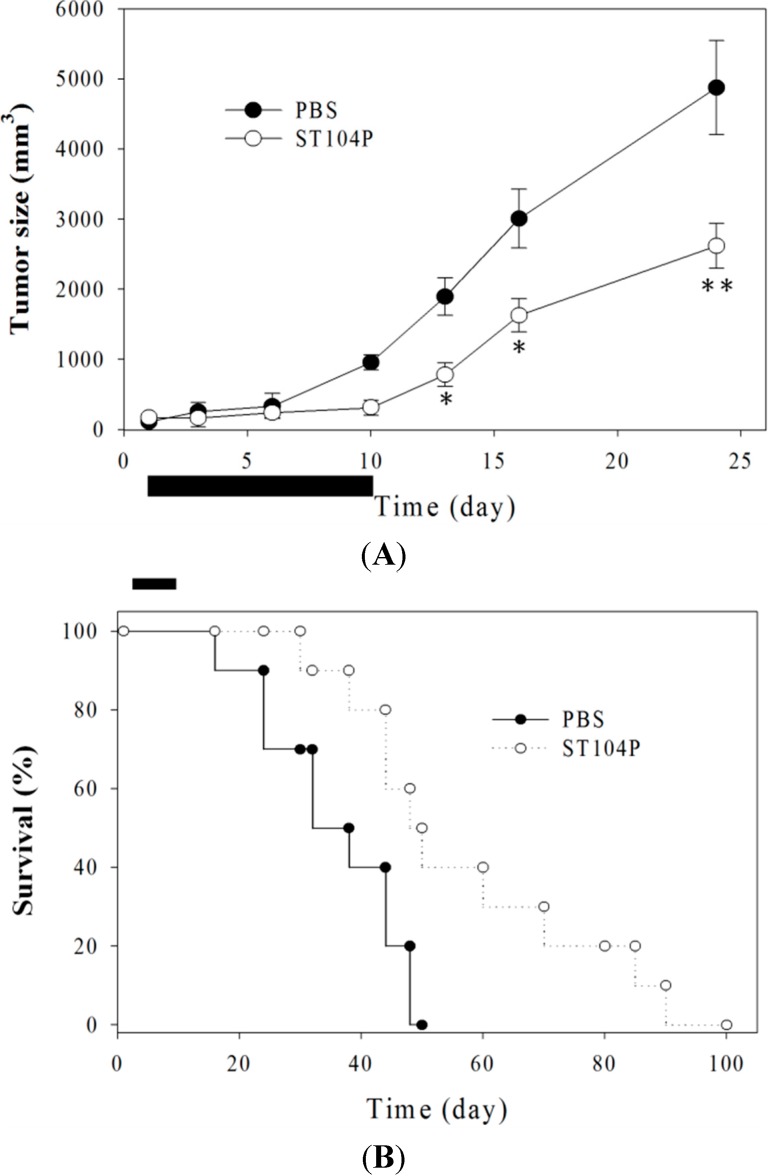
Injection of ST104P suppressed tumor growth and prolonged the survival of Lewis lung carcinoma in mice. (**A**) The subcutaneous dorsa of mice were implanted with Lewis lung carcinomas. The tumor sizes in mice during treatment with ST104P or control were recorded. Treatment was performed by intratumor injection of ST104P (100 µg in 0.1 mL PBS) daily from day 0 to day 10 as indicated by the horizontal bar. Data were mean ± SEM for ten mice. Asterisks indicate statistical significance *versus* control. * *p* ˂ 0.05 and ** *p* ˂ 0.01; and (**B**) The overall survival rates of PBS- or ST104P-treated mice (*n* = 10). Kaplan-Meier survival analysis indicated the ST104P treatment significantly enhanced the survival rates of tumor-bearing mice (*p* < 0.001). This experiment was repeated with comparable results.

To further validate the anti-angiogenic activities of ST104P, we treated established Lewis lung carcinoma grown in syngeneic C57BL/6J mice by periodic injection of ST104P. The growth of Lewis lung carcinoma was significantly perturbed by ST104P treatment; the average tumor size of ST104P-treated mice (2620 ± 320 mm^3^) was significantly smaller (about 40% decrease) than that of saline-treated groups (4876 ± 670 mm^3^; *p* < 0.05; Figure 3A). Histological analysis showed that the number of CD31-postive neovascularized vessels was significantly reduced in ST104P-treated tumors compared with control (data not shown). Above all, mice treated with ST104P survived significantly longer than animals of the vehicle-treated group (*p* < 0.01; Figure 3B). There was no evident weight loss or adverse effects in mice treated with ST104P, suggesting that ST104P injection was well tolerated by animals. Together, these results indicate that ST104P may be applicable to cancer therapy.

### 2.4. ST104P Attenuated Migration and Tube Formation of Cultured Endothelial Cells

**Figure 4:**
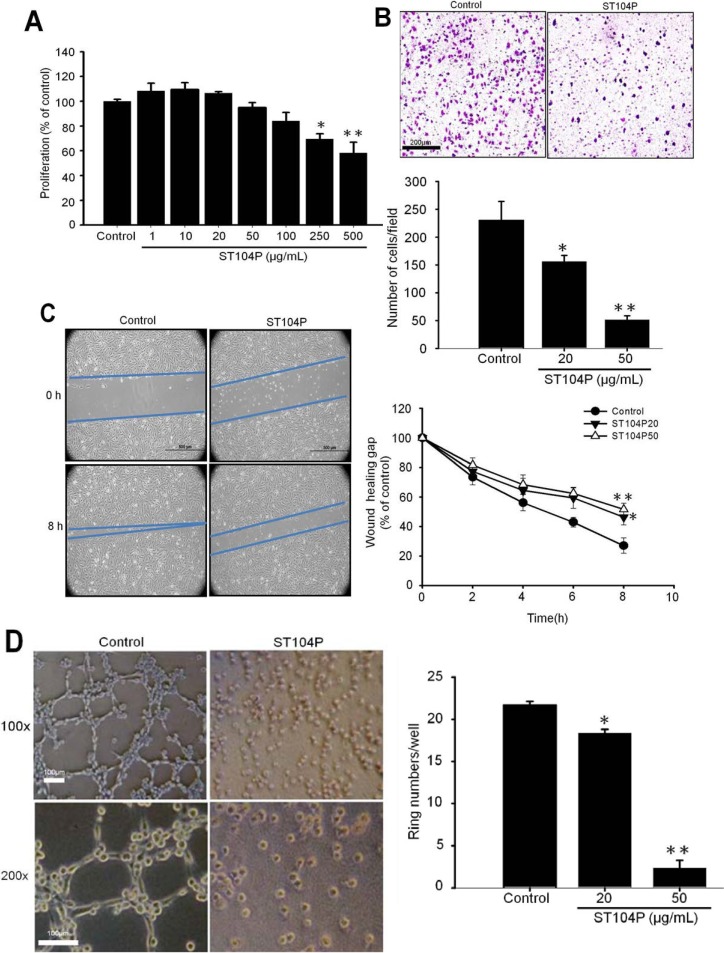
Effect of ST104P on various angiogenic processes in cultured endothelial cells. (**A**) Effect of ST104P on proliferation of human umbilical endothelial cells (HUVECs)MTT assay was performed to determine the viability of HUVECs after treatment with ST104P (1–500 μg/mL) for 48 h; (**B**) Effect of ST104P on migration of endothelial cells. By using trans-well migration assay, the number of migrated HUVECs after treatment with ST104P (20 and 50 μg/mL) for 8 h were quantified by image analysis taken from five different low-power fields (100× magnification; scale bar = 100 µm); (**C**) Effect of ST104P on wound healing of endothelial cells. After scratch wound, the extent of wound closure in HUVECs with or without ST104P (20 and 50 μg/mL) was quantified by image analysis from 0–8 h of incubation (50× magnification; scale bars = 500 µm). The area of wound healing of endothelial cells was analyzed statistically; and (**D**) Effect of ST104P on tube formation of endothelial cells. By plating on matrigel-coated plate, the capillary-like networks of HUVECsafter treatment with ST104P (20 and 50 μg/mL) for 8 h were monitored and recorded by light microscopy (100× and 200× magnification, scale bar = 100 µm). Data were mean ± SEM of triplicate experiments. Asterisks indicate statistical significance *versus* control. * *p* ˂ 0.05 and ** *p* ˂ 0.01.

To delineate the influence of ST104P on the distinct angiogenic processes, we subsequently investigated the effect of ST104P on proliferation, migration, tube formation and wound healing in cultured endothelial cells. By using 3-(4,5-dimethylthiazol-2-yl)-2,5-diphenyl-tetrazolium bromide (MTT) assay, it was found that ST104P exerted marginal influence on the proliferation of cultured endothelial cells (less than 30% at 500 µg/mL; Figure 4A).

Cell migration is essential for angiogenesis in endothelial cells [21]. To identify the effects of ST104P on cell migration, we used two types of migration models: a trans-well Boyden chamber migration assay and a wound-healing migration assay. In the Boyden chamber migration assay, ST104P dose-dependently inhibited the migration of endothelial cells with a half maximal inhibitory concentration (IC_50_) between 20–50 μg/mL (Figure 4B). Likewise, application of ST104P (50 μg/mL) effectively attenuated the healing of scratch wound in endothelial cells (Figure 4C).

Tube formation by interaction of endothelial cells with extracellular matrix is also critical for angiogenesis [22]. By using Matrigel assay, it was observed that ST104P (50 μg/mL) potently perturbed the tube formation of endothelial cells by about 80% of control (Figure 4D). These results reveal that ST104P interferes with multiple steps during angiogenesis in endothelial cells.

### 2.5. ST104P Inhibited Secretion and Expression of Matrix Metalloproteinase-2 (MMP-2) in Endothelial Cells

**Figure 5:**
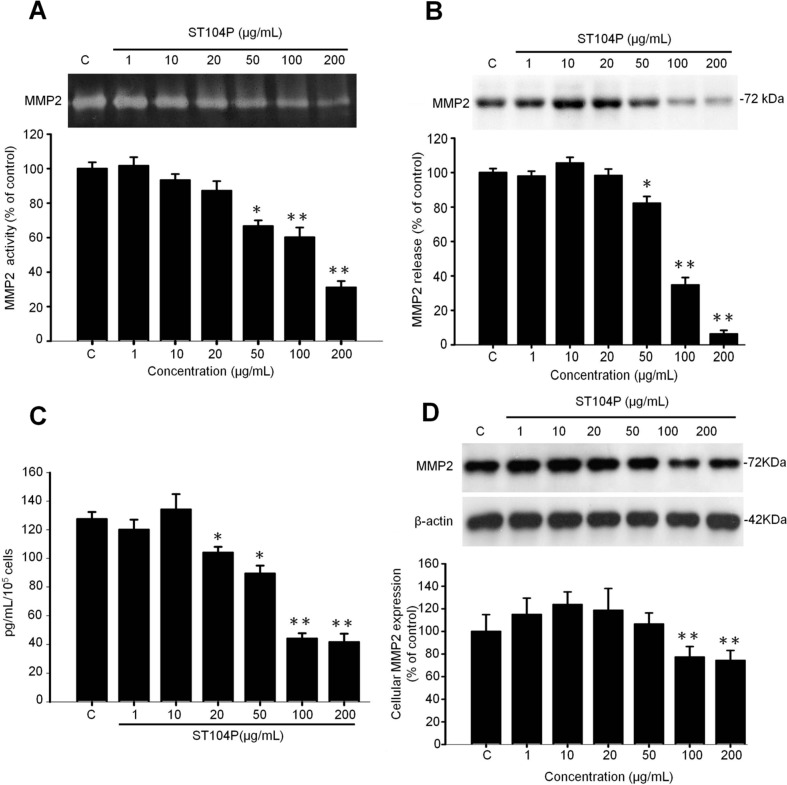
Effect of ST104P on matrix metalloproteinases (MMPs) expression in endothelial cells. HUVEC were treated with various concentration of ST104P (1–200 μg/mL) for 24 h, the condition media and pellet were collected and performed to determine the MMPs expression in HUVECs. (**A**) MMP-2 activities were analyzed by gelatin zymography, gelatinolytic activities of MMP-2 (72 kDa) were indicated by clear bands in the zymograms with viability cell amount serving as a loading control. To evaluate MMP-2 secretion from HUVECs in culture medium, MMP-2 expression was tested by Western blot (**B**) and ELISA analysis (**C**), after normalization based on viability cell proteins; and (**D**) The expression of cytosolic MMP-2 was determined by Western blot and normalized with β-actin level. Data were mean ± SEM of triplicates. Asterisks indicate statistical significance *versus* control. * *p* ˂ 0.05 and ** *p* ˂ 0.01.

Matrix metalloproteinases (MMPs) constitute a large family of extracellular matrix degrading proteases and are involved in a number of physiological and pathological processes including angiogenesis [14,23]. Among them, MMP-2 and MMP-9 are gelatinases and frequently investigated for their roles in angiogenesis and tumorigenesis [7,15,24]. By using gelatin-zymography analysis, it was shown that ST104P potently and dose-dependently suppressed the MMP-2 activities of endothelial cells (Figure 5A). Immunoblot analysis (Figure 5B) and ELISA (Figure 5C) indicated the suppressed MMP-2 activities were due to the reduced MMP-2 release by ST104P treatment. Finally, immunoblot analysis revealed the attenuated MMP-2 release by ST104P was correlated with the decreased cellular MMP-2 protein levels in endothelial cells (Figure 5D). Therefore, ST104P is a potent inhibitor of MMP-2 secretion in endothelial cells through down-regulation of MMP-2.

## 3. Discussion

The present study unveils for the first time that ST104P, a polysulfated, cyclic compound, exhibits anti-angiogenic functions *in vitro* and *in vivo*. ST104P shows excellent water solubility while potently inhibiting the secretion of MMP-2 by endothelial cells. Besides, ST104P also perturbed the migration and tube formation of endothelial cells. Finally, repeated administration of ST104P into Lewis lung carcinoma resulted in delayed tumor growth and prolonged the life span of tumor-bearing mice. In addition to anti-viral and anti-thrombotic activities [17,18,19], the anti-angiogenesis properties of ST104P may be useful for treatment of cancer and other diseases due to excessive angiogenesis. One well known example of the multiple functions of an anti-angiogenic compound is thalidomide [25], which was initially used in pregnant women to prevent morning sickness and later stopped as it caused abnormalities in infants. However, thalidomide has now been applied for cancer therapy after discovery of its anti-angiogenesis function.

Unlike other angiogenesis inhibitors such as TNP-470 [26], angiostatin [27] or endostatin [9], ST104P exhibits marginal cytotoxicity on endothelial cells. Even at the highest concentration tested (500 μg/mL), the ST104P-induced growth inhibition was less than 30% in cultured endothelial cells. In contrast, the dose required for ST104P to elicit angiogenesis blockade was far lower. For example, the IC_50_ for ST104P to inhibit angiogenesis steps, including migration, tube formation and MMP secretion, was about 50 μg/mL (equivalent to 0.034 nM). Besides, ST104P is highly soluble and could be formulated with saline or other buffer systems. The above features of high potency, low toxicity and excellent water solubility warrant the further clinical development of ST104P.

The present study indicates the role of MMP-2 down-regulation and endothelial migration in ST104P-induced angiogenesis inhibition. Since the breakdown of the extracellular barrier by MMPs is the first step during neovascularization, it is rational to attribute the attenuated migration of endothelial cells to reduced MMP-2 secretion by ST104P. However, it remains unclear whether an additional pathway(s) participates in ST104P-mediated angiogenesis inhibition. This may require further studies. Recently, human clinical trials on MMP inhibitors were completed and yielded differential outcomes [28]. Given the high potency in halting MMP secretion, ST104P may deserve future investigations based on its indication as an MMP inhibitor.

One important finding of the present study is the therapeutic efficacy of ST104P in delaying tumor growth and extending the survival rate in mice bearing Lewis lung carcinoma. Histological analysis indicates the role of angiogenesis blockade in ST104P-mediated tumor suppression. Because angiogenesis is essential for growth as well as metastasis in most types of cancer, ST104P may be applicable for treatment of various types of cancer including the metastatic ones. Indeed, pilot studies from our group suggest that ST104P therapy is also effective in perturbing the progression of primary B16-F10 melanoma as well as lung-metastatic B16-F10 melanoma in mice (data not shown). Interestingly, despite of its low cytotoxicity, ST104P might exert a negative influence on the oncogenic behaviors of tumors cells, such as invasion and anchorage-independent growth. Further studies are warranted to investigate the anti-neoplastic mechanism of ST104P in tumor and non-tumor cells.

## 4. Experimental Section

### 4.1. Chemical and Antibodies

ST104P (4,5-dihydroxynaphthalene-2,7-disulfonic acid) was provided by Su-Ying Liu (Sagittarius Life Science Corp., Taipei, Taiwan) and dissolved in phosphate-buffered saline (PBS). Antibodies against MMP-2 and β-actin were obtained from Santa Cruz Biotechnology, Inc. (Santa Cruz, CA, USA).

### 4.2. Rat Aortic Ring Assay

The *ex vivo* angiogenesis assay was performed as previously described [29,30]. Thoracic aortas were removed from Sprague-Dawley rats (male; 8-week-old) and immediately transferred to a culture dish containing ice-cold serum-free MCDB131 media (Life technologies Ltd., Paisley, Scotland). The peri-aortic fibroadipose tissue was carefully removed with fine microdissecting forceps and iridectomy scissors, paying special attention not to damage the aortic wall. One-millimeter long aortic rings (approximately 15 per aorta) were sectioned and extensively rinsed in five subsequent washes of MCDB131 media. Ring-shaped explants of aorta were then embedded in the 1 mL mixtures of Matrigel and MCDB131 (1:1). Then, the aortic rings were polymerized and kept in triplicate at 37 °C in 24-well culture plates. After polymerization, each well was added with 1 mL of MCDB131 (Life technologies Ltd., Paisley, Scotland) supplemented with 25 mM NaHCO_3_, 2.5% rat serum, 1% glutamine, 100 U/mL penicillin, 100 μg/mL streptomycin and PBS or ST104P at 1–100 μg/mL to the upper on Matrigel-based embedded aortic ring. The rings were kept at 37 °C in a humidified environment for 7 days and the vascular sprouting was examined by microscope equipped with digital images system (Olympus; Tokyo, Japan). The greatest distance from the aortic ring body to the end of the vascular sprouts (sprout length) was measured by National Institutes of Health (NIH) Image program at sixteen distinct points per ring.

### 4.3. Zebrafish Angiogenesis Model

Zebrafish (*Daniorerio*) transgenic lines *Tg(kdrl:mCherry)^ci5^* and *Tg(fli1a:negfp)^y7^* [31,32] were from Taiwan Zebrafish Core Facility (Academia Sinica, Taipei, Taiwan) and maintained at the 28.5 °C incubator with approval by the Animal Care Committee at National Sun Yat-sen University (Kaohsiung, Taiwan). Embryos were treated with 0.003% 1-phenyl-2-thiourea (PTU; Sigma, St. Louis, MO, USA) at 6 h post fertilization (hpf) to prevent pigment formation and used to monitor the effects of ST104P on embryonic angiogenesis [33]. Zebrafish embryos were generated by natural pair-wise mating and raised at 28 °C in embryo water (0.2 g/L of Instant Ocean Salt in distilled water). Approximately 20 healthy embryos were placed in 6 cm dishes and various concentrations of ST104P were separately added into embryo water at 6 h post fertilization (hpf). The embryo water containing ST104P was replaced daily. At 30 hpf, the embryos were anesthetized using 0.05% 2-phenoxyethanol in embryo water and examined for vessel development, especially in the intersegmental vessels (ISV) and the caudal vein plexus (CVP), at 48 hpf observed migration and proliferation of endothelial cells in the ISV, using a fluorescent microscope with digital images system (Olympus; Tokyo, Japan). The length of the ISV was measured by NIH Image program.

### 4.4. Cells Cultures

Human umbilical vein endothelial cells (HUVECs) were purchased (LONZA ,Walkersville, MD, USA) and cultured in M199 medium (Life Technologies, Gaithersburg, MD, USA) containing 15% fetal bovine serum (FBS) (HyClone, Thermo scientific, Logan, UT, USA), 20 U/mL porcine heparin (Sigma) and 100 μg/mL endothelial cell growth supplement (Calbiochem; La Jolla, CA, USA) as previously described [34]. HUVECs were used for all the experiments at passages 2–7. These cells were incubated under humidified conditions in 95% air and 5% CO_2_ at 37 °C.

### 4.5. Western Blot Analysis

The protein extracts from HUVECs were prepared using PRO-PRE protein extraction solution (iNtRON Biotechnology Inc, Kyungki-Do, Korea). An aliquot of proteins was separated by sodium dodecyl sulfate-polyacrylamide gel (SDS-PAGE) and transferred onto the polyvinylidene difluoride membranes (PVDF) (Millipore, Bedford, MA, USA). After blocking for 30 min used 5% fat-free milk in Tris-Buffered Saline Tween-20 (TBST), the membrane was incubated with primary antibodies for 2 h at room temperature, and then bound with horseradish peroxidase (HRP)-conjugated secondary antibodies (anti-mouse, anti-rabbit antibodies, Santa Cruz, CA, USA; 1:5000 dilution) for 1 h. Immunoreactivity was detected by emitter-coupled logic (ECL) plus chemiluminescence kit (Amersham Biosciences, Piscataway, NJ, USA), and the membrane was expose to X-ray film for autoradiogram. Intensities of the immune bandings were quantified by densitometric scanning. The primary antibodies used in this study were antibodies against MMP-2 (1:1000 dilutions; Santa Cruz Inc., CA, USA) and β-actin (1:5000 dilution; Santa Cruz Inc., CA, USA).

### 4.6. Gelatin Zymography

The MMPs expression of ST104P-treated endothelial cells was measured by gelatin zymography. The secretion of MMPs was assessed by 0.1% gelatin-sodium dodecyl sulfate (SDS) polyacrylamide gel electrophoresis (PAGE) zymography. Briefly, HUVECs at near 80% confluence were supplemented with serum-free M199 media and treated with PBS or ST104P at 1–200 μg/mL for 24 h. Aliquots of conditioned media were subjected to separation with 10% SDS-PAGE containing 0.1% type-A gelatin (Sigma Chemical Co., St. Louis, MO, USA). After electrophoresis, the gel was washed twice with 2.5% Triton X-100, incubated in buffer containing 40 mM Tris-HCl, pH 8.0; 10 mM CaCl_2_ and 0.01% sodium azide at 37 °C for 18–24 h, stained with 0.25% Coomassie blue R-250 in 50% methanol and 10% acetic acid for 1 h, and destained with 10% acetic acid and 20% methanol. The gelatinolytic regions by MMPs were visualized as clear bands in the zymograms and quantified by densitometer.

### 4.7. Enzyme-Linked Immunosorbent Assay (ELISA)

The MMP-2 concentrations in cultured media of HUVECs were measured by MMP-2 ELISA kit (R&D systems, Inc., Minneapolis, MN, USA) following protocols provided by the manufacturer.

### 4.8. Cell Proliferation Assay

Cell viability was measured by a quantitative colorimetric assay with 3-(4,5-dimethylthiazol-2-yl)-2,5-diphenyl-tetrazolium bromide (MTT) assay. HUVECs were cultured in a collagen-coated 96-well plate at density of 5 × 10^3^ cells/mL overnight. After 48 h, the cells were treated with PBS or ST104P at 1–500 μg/mL in 10% FBS serum medium, then washed and incubated in M199 medium containing 0.5 mg/mL of MTT for 2 h at 37 °C. The formazan in viable cells was dissolved with dimethylsulfoxide (DMSO) (Sigma-Aldrich, Co., St. Louis, MO, USA) and determined by reading optical densities with a microplate reader (BIO-RAD Benchmrk plus, France) at an absorption wavelength of 570 nm.

### 4.9. Migration Assay

The cell migration assay was performed as previously described [30,35]. HUVECs were seeded in the upper compartment of the chamber (2.5 × 10^4^ cells/50 µL per well) and supplemented with M199 media containing PBS or various doses of ST104P (1–200 μg/mL). The lower compartment was filled with 30 µL of M199 media containing 10% FBS media. A polycarbonate filter (8 µm pore size Nucleopore; Costar, Cambridge, MA, USA) coated with 0.1% gelatin to allow cell adhesion was used to separate the compartments. After incubation for 8–10 h in a humidified 5% CO_2_ atmosphere chamber at 37 °C, cells on the upper side of the filter were allowed to move to the lower side. Migrated cells were fixed in absolute methanol and stained with 10% Giemsa solution (Merck, Darmstadt, Germany). Finally, the fixed cells were photographed by microscope with digital images system (Olympus; Tokyo, Japan), and counted as mean ± SEM number of cells per filter under five different low-power fields.

### 4.10. Tube Formation Assay

The tube formation assay was performed as previously described [30,35]. Matrigel (Becton Dickinson, Bedford, MA, USA) was diluted with cold M199 serum-free media in 1:1 ratio. The diluted Matrigel solution was added to 96-well plates (70 µL per well) and allowed to form a gel at 37 °C for 1 h. Subsequently, cell suspensions (3 × 10^4^ cells in 70 μL per well in M199 media containing 10% FBS with ST104P at 1–200 μg/mL) were plated on Matrigel-coated wells and incubated for an additional 6–8 h at 37 °C in 5% CO_2_. Afterwards, the endothelial tubes were observed and photographed by microscope with a digital images system (Olympus; Tokyo, Japan).

### 4.11. Scratch Wound Healing Assay

The migration of endothelial cells was assessed using a scratch migration assay as described previously [36]. Briefly, a gap of approximately 1 mm was created in the adherent layer of confluent endothelial cells (in six-well plates) by introducing a scratch using a sterile pipette tip (Gilson, Inc., Middleton, WI, USA). After treatment with ST104P (1–200 μg/mL), the extent of gap closure was measured by a microscope with a digital images system (Olympus; Tokyo, Japan) at different time intervals with the NIH Image program.

### 4.12. Animal Studies

Animal study was carried out in the animal facility of Kaohsiung Veterans General Hospital in accordance with institutional guidelines. Male C57BL/6J mice (6–8 weeks-old; Animal Center of Kaohsiung Veterans General Hospital, Taiwan) were used. Mice were acclimated and caged in groups of four or less. All mice were fed with a diet of animal chow and water *ad libitum*. Animals were anesthetized in a methoxyflurane chamber prior to all procedures and were observed until fully recovered. Animals were sacrificed by a lethal dose of methoxyflurane.

### 4.13. Treatment of Lewis Lung Carcinoma

C57BL/6J mice were implanted with Lewis lung carcinomas cells using the techniques described previously [9,37]. Briefly, the LL2 Lewis lung carcinoma cells were resuspended in PBS at 2.5 × 10^6^ cells/mL and injected into C57B16/J mice (*n* = 20) with 0.1 mL of the cell suspension to induce tumor. Tumors were measured with a dial-caliper and volumes were determined using the formula width^2^ × length × 0.52. After tumor volumes reached 100–200 mm^3^ (day 0), mice were randomized into two groups receiving periodic injection of ST104P (*n* = 10; 100 μg in 0.1 mL saline per mice on day 1, 3, 6 and 10) or saline (*n* = 10) by subcutaneous injection at a site near tumor. The measurement of tumor size was concluded when mice began to die (day 24). In addition, the survival rate of mice in different groups was recorded and analyzed by Kaplan-Mier Survival analysis.

### 4.14. Statistical Analysis

All data are expressed as means ± SEM. Paired *t*-test was used to statistically assess the differences between the groups. The differences were considered significant with *p* value lee than 0.05 (* *p* ˂ 0.05 and ** *p* ˂ 0.01).

## 5. Conclusions

ST104P is a novel angiogenesis inhibitor that functions at various angiogenic stages including MMPs secretion without overt toxicity. Besides, ST104P therapy perturbs tumor growth and prolongs the survival of tumor-bearing mice. Thus, ST104P may be useful for treatment of cancer and other angiogenesis-dependent diseases.
